# Plantaricin NC8 αβ rapidly and efficiently inhibits flaviviruses and SARS-CoV-2 by disrupting their envelopes

**DOI:** 10.1371/journal.pone.0278419

**Published:** 2022-11-30

**Authors:** Abubakr A. M. Omer, Jorma Hinkula, Pham-Tue-Hung Tran, Wessam Melik, Elisa Zattarin, Daniel Aili, Robert Selegård, Torbjörn Bengtsson, Hazem Khalaf

**Affiliations:** 1 School of Medical Sciences, Faculty of Medicine and Health, Örebro University, Örebro, Sweden; 2 Department of Biomedical and Clinical Sciences (BKV), Division of Molecular Medicine and Virology, Mucosa infection och inflammation Center (MIIC), Linköping University, Linköping, Sweden; 3 Laboratory of Molecular Materials, Department of Physics, Chemistry and Biology (IFM), Division of Biophysics and Bioengineering, Linköping University, Linköping, Sweden; Consejo Superior de Investigaciones Cientificas, SPAIN

## Abstract

Potent broad-spectrum antiviral agents are urgently needed to combat existing and emerging viral infections. This is particularly important considering that vaccine development is a costly and time consuming process and that viruses constantly mutate and render the vaccine ineffective. Antimicrobial peptides (AMP), such as bacteriocins, are attractive candidates as antiviral agents against enveloped viruses. One of these bacteriocins is PLNC8 αβ, which consists of amphipathic peptides with positive net charges that display high affinity for negatively charged pathogen membrane structures, including phosphatidylserine rich lipid membranes of viral envelopes. Due to the morphological and physiological differences between viral envelopes and host cell plasma membranes, PLNC8 αβ is thought to have high safety profile by specifically targeting viral envelopes without effecting host cell membranes. In this study, we have tested the antiviral effects of PLNC8 αβ against the flaviviruses Langat and Kunjin, coronavirus SARS-CoV-2, influenza A virus (IAV), and human immunodeficiency virus-1 (HIV-1). The concentration of PLNC8 αβ that is required to eliminate all the infective virus particles is in the range of nanomolar (nM) to micromolar (μM), which is surprisingly efficient considering the high content of cholesterol (8–35%) in their lipid envelopes. We found that viruses replicating in the endoplasmic reticulum (ER)/Golgi complex, e.g. SARS-CoV-2 and flaviviruses, are considerably more susceptible to PLNC8 αβ, compared to viruses that acquire their lipid envelope from the plasma membrane, such as IAV and HIV-1. Development of novel broad-spectrum antiviral agents can significantly benefit human health by rapidly and efficiently eliminating infectious virions and thereby limit virus dissemination and spreading between individuals. PLNC8 αβ can potentially be developed into an effective and safe antiviral agent that targets the lipid compartments of viral envelopes of extracellular virions, more or less independent of virus antigenic mutations, which faces many antiviral drugs and vaccines.

## Introduction

Emerging viral pathogens, including SARS-CoV-2 causing the current pandemic, show an increased frequency and underscores our limited therapeutic resources against viral infections. Most available antiviral agents target different stages in the virus life cycle and their clinical use is limited primarily by resistance and toxicity [1]. While there are many approved antiviral drugs for the treatment of some viruses, such as HIV-1 [2, 3], there are no licensed prophylactic or therapeutic drugs for the majority of other enveloped viruses [4]. Development of novel antiviral compounds against large classes of viruses, as opposed to single-pathogen therapeutics, would be more advantageous by enabling treatment of a wide range of viral infections [5, 6]. This is particularly interesting considering that vaccine development is a costly and time-consuming process and, most importantly, the produced vaccines may not provide long-lasting robust immune responses against the rapidly mutating viruses, such as influenza and coronaviruses [7–9]. Consequently, a potent broadspectrum antiviral drug would have great clinical and public health importance by combating both existing and emerging human virus pathogens.

Antiviral subtsances based on compounds that directly destroy the virions, such as antimicrobial peptides (AMPs) [5, 10], would rapidly eliminate the viruses and their subsequent pathogenesis, and thereby limit their dissemination to other organs and spread between individuals. Bacteriocins are small AMPs produced by bacteria, including lactobacilli, that kill microbes usually by membrane disruption [11, 12]. Bacteriocins have become attractive candidates for therapeutic applications in traditional medicine against infections due to their high potency, broad-spectrum activity and beneficial effects on tissues [13–15]. Plantaricin NC8 (PLNC8) αβ is a two-peptide bacteriocin expressed by *Lactobacillus plantarum* strains. PLNC8 αβ consists of amphipathic peptides with positive net charges that display high affinity for negatively charged lipid membranes and pathogen membrane structures. This interaction causes rapid disruption of the bacterial membrane integrity, ultimately leading to loss of homeostasis and killing of the target pathogen. We have previously reported that PLNC8 αβ effectively inhibits and kills several bacterial pathogens, including *Staphylococcus* spp., and markedly enhances the effects of antibiotics [16].

Enveloped viruses, such as influenza viruses, coronaviruses, filoviruses, and flaviviruses cover their protein capsid with a lipid envelope through budding from host cell membranes [4]. For many virus families, e.g. *Coronaviridae* and *Flaviviridae*, the process of translation, replication, assembly, and budding occur in the ER/Golgi apparatus to produce mature viruses, which are then released through exocytosis [17]. The lipid envelope of these viruses is thus derived from the ER. The lipid composition of the ER membrane differs from that of the plasma membrane, e.g. by an equal distribution of anionic lipids (for example phosphatidylserine) in both leaflets of the ER membrane, while the plasma membrane is assymetric in which all the charged lipids are oriented towards the cytosol [18]. Moreover, the high cholesterol content in the plasma membrane (~35%) contributes to membrane stability and fluidity, and prevents membrane disruption by PLNC8 αβ and other similar membrane active peptides. The ER membrane also contains some cholesterol (~8%) that provides stability, however, expresses limited resistance against membrane-active amphipathic peptides [18, 19]. Consequently, the morphological and physiological differences between the viral envelope and host cell membranes make viral membranes ideal targets for antiviral therapy by using antimicrobial peptides such as PLNC8 αβ. In addition, we have previously shown that PLNC8 αβ displays no cytotoxicity towards human cells, but rather induces cell proliferation and expression of several growth factors, including TGF-β1, IGF-1 and EGF [20]. PLNC8 αβ is thus an interesting candidate as antiviral agents against enveloped viruses.

We hypothesize that PLNC8 αβ inhibits enveloped viruses by disrupting their lipid bilayers and that the extent of antiviral effects is dependent on the cellular origin and chemical composition of the envelope. In this work, we have tested and verified this hypothesis through experimental microbiological studies by investigating the effects of PLNC8 αβ against various viral species obtaining their envelopes from different cellular compartments.

## Materials and methods

### Cell culture

Monkey (*Cercopithecus aethiops*) epithelial kidney cells (Vero E6, ATCC, CRL-1586), dog (*Canis familiaris*) epithelial kidney cells (MDCK, ATCC, CCL-34), and human lung epithelial cells (A549, ATCC CCL-185) were maintained in Dulbecco’s Modified Eagle’s Medium (DMEM) containing 1 g/L glucose (Gibco), supplemented with 10% heat-inactivated fetal bovine serum (HI‒FBS, Gibco) and 100 U/mL penicillin-streptomycin (PEST, Gibco) at 37°C in 95% air and 5% CO_2_. Jurkat T-cells cells (ATCC, TIB-152) were maintained in RPMI 1640 medium (Fisher scientific, Austria) with 1.5 mM *L*-glutamine (Invitrogen, USA) and supplemented with 10% FBS. PBMC were isolated by the density gradient medium Ficoll-Paque^TM^ Plus (Amersham Biosciences, Sweden) according to the manufacturers’ instructions.

### Virus strains and propagation

The following virus strains were used in this study: Langat virus strain TP21 sequence (LGTV, accession number NC003690), West nile virus Kunjin strain (KUNV, accession number AY274504), HIV-1 (subtype B, MN strain), IAV (H1N1/CApdm09), and Severe Acute Respiratory Syndrome Coronavirus 2 (β-SARS-CoV-2). All virus strains were propagated in Vero cells at a MOI of 0.1, while HIV-1/MN that were propagated in Jurkat T-cells [21, 22] at a MOI of 0.1 (or 100 TCID/0.1 ml).

### Peptides

**Fig 1:**
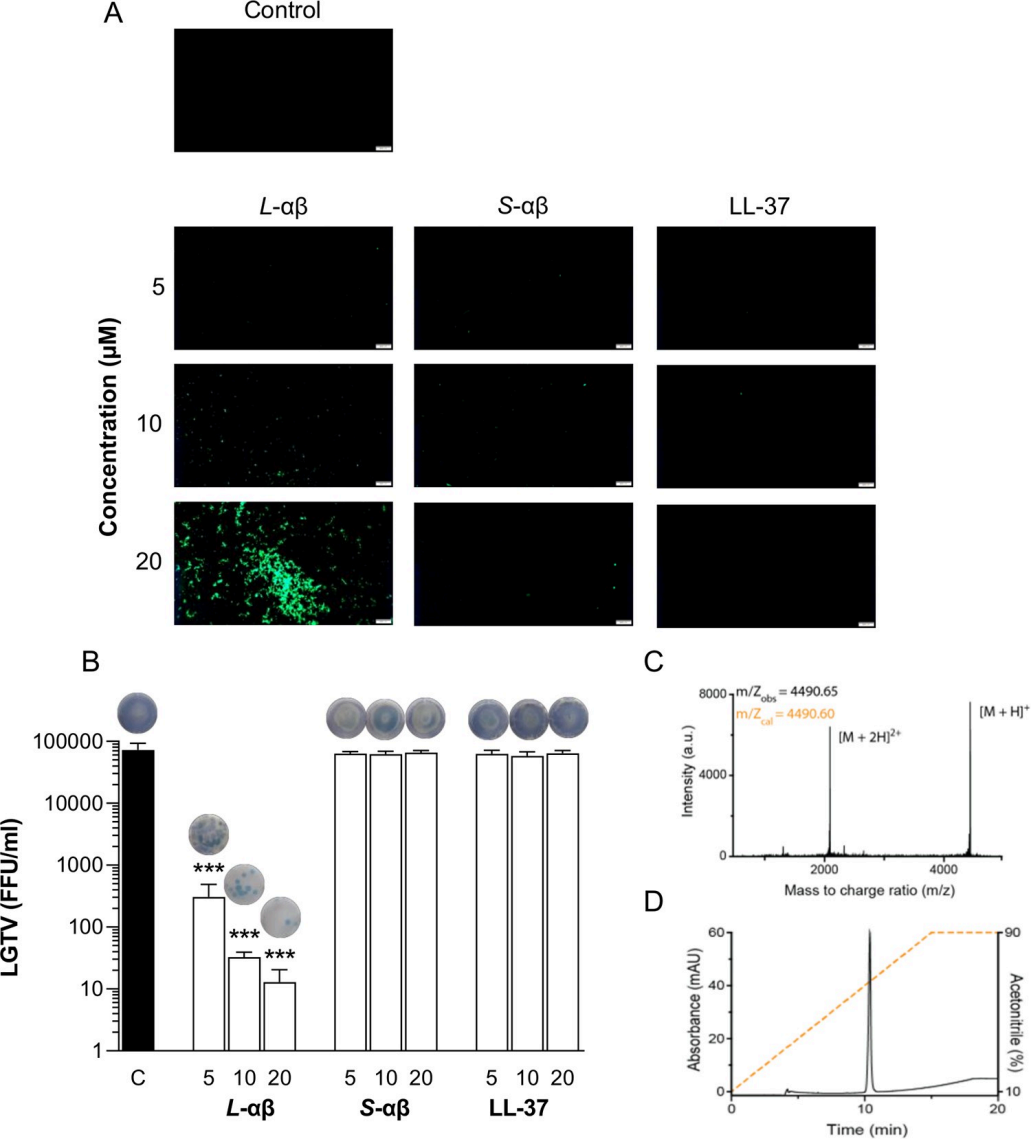
PLNC8 αβ, but not scrambled peptide or LL-37, is effective against flaviviruses. LGTV (1×10^5^), suspended in DMEM, were either left untreated or exposed to *L*-PLNC8 αβ (L-αβ, 1:1), *S*-PLNC8 αβ (S-αβ, 1:1) or LL-37 at a final concentration of 5, 10 and 20 μM. **A-** The samples were fixed with 4% PFA for 2 h followed by addition of Sytox Green for 5 min, and images were captured with Olympus BX41 at 40× magnification. Scale bar is 200 μm. **B-** LGTV was incubated in cell culture media containing the indicated concentrations of *L*-PLNC8 αβ, *S*-PLNC8 αβ or LL-37 for 1 h at 37°C. The suspension was then added to Vero cells and incubated for 1 h. An overlay media was casted onto the cells and the plates were incubated for 3 days. Viral load was quantified by performing immunofocus-based plaque assay. The indicated concentrations are in μM. Representative images of the virus plaques are presented above the bars. The virus particles are rapidly permeabilized by *L*-PLNC8 αβ, forming large aggregates, and decreased the viral load by >99.9%. **C**- Peptide identity of the purified LL-37 was analyzed and confirmed with MALDI-ToF MS. **D**- The purity of LL-37 was confirmed by HPLC yielding a single peak. Orange dotted line indicates gradient of acetonitrile (10–90%) in H_2_O containing 0.1% TFA. The data (n = 4) are presented as mean with SEM and analysed using one-way ANOVA with Dunnett´s multiple comparison test (***p<0.001).

The peptides *L*-PLNC8 αβ and *D*-PLNC8 αβ were purchased from GL Biochem Ltd (Shanghai, China). Synthesis of scrambled *L*-PLNC8 αβ has been described previously [16]. LL-37 was synthesized in 100 μM scale on a microwave assisted automated peptide synthesizer (Liberty Blue, CEM, Matthews, USA) using ProTide Rinkamide (LL) as resin. Fmoc protected amino acids were utilized and sequentially coupled under microwave conditions (90°C, 2 min) to the growing peptide using a fivefold excess of amino acids, Oxyma as base and DIC as coupling reagent. N-terminal Fmoc-deprotection was achieved by treatment with 20% piperidine in DMF under microwave conditions (90°C, 2 min). After final Fmoc deprotection, the crude peptide was cleaved from its solid support by treatment with trifluoracetic acid (TFA): triisopropylsilane: H_2_O (95:2.5:2.5) for 3 h before being concentrated, precipitated twice with cold diethyl ether and dried. The crude LL-37 was purified using a reversed phase C-18 column (ReproSil Gold) attached to a semi-preparative HPLC system (Dionex Ultimate 3000, Thermo Fisher Scientific, Waltham, USA). Peptide identity and purity was confirmed using MALDI-TOF MS and HPLC (Fig 1C and 1D). The amino acid sequences, molecular weight, and net charge at pH 7 of all peptides are presented in Table 1. In the peptides denoted *L*-PLNC8 α and *L*-PLNC8 β, all amino acid residues are in the *L*-configuration, and in the peptides denoted *D*-PLNC8 α and *D*-PLNC8 β, all amino acid residues are in the *D*-configuration. Scrambled forms of PLNC8 α and PLNC8 β (denoted *S*-PLNC8 α and *S*-PLNC8 β, respectively) were generated by randomly displacing the amino acids of the original sequences to show that the order of the amino acids is important for the activity of PLNC8 α and PLNC8 β. The human cathelicidin-derived peptide LL-37 is a known antimicrobial peptide that was used as a control.

**Table 1:** Amino acid sequence, molecular weight, and net charge at pH 7 of the peptides *L*-PLNC8 αβ [11], *D*-PLNC8 αβ, *S*-PLNC8 αβ [16], and LL-37 that were used in this study.

Name	Sequence	MW (g/mol)	Net charge at pH 7
*L*-PLNC8 α	DLTTKLWSSWGYYLGKKARWNLKHPYVQF	3587	+4.1
*D*-PLNC8 α	DLTTKLWSSWGYYLGKKARWNLKHPYVQF	3587	+4.1
*S*-PLNC8 α	TWLKYGHGDAKLWSWSKPLNLTFRYQYRK	3587	+4.1
*L*-PLNC8 β	SVPTSVYTLGIKILWSAYKHRKTIEKSFNKGFYH	4001	+5.2
*D*-PLNC8 β	SVPTSVYTLGIKILWSAYKHRKTIEKSFNKGFYH	4001	+5.2
*S*-PLNC8 β	LKLWNTYGTFSRFYTSKSEVKIAHGIKSIHVPYK	4001	+5.2
LL-37	LLGDFFRKSKEKIGKEFKRIVQRIKDFLRNLVPRTES	4493	+6.0

### Liposome preparation

Liposomes were prepared by thin-film hydration followed by extrusion [23]. Stock solutions of the lipids 1-palmitoyl-2-oleoyl-sn-glycero3-phosphatidylcholine (POPC), 1-palmitoyl-2-oleoyl-sn-glycero-3-phosphoL-serine (POPS) and cholesterol (Chol) were mixed at molar ratio 95:0:5, 65:30:5, 70:0:30, and 81:11:8. The solvent was slowly evaporated via nitrogen stream, then the vials were dried in a vacuum desiccator overnight. Dry films were hydrated by addition of 1 ml 5(6)-carboxyfluorescein (CF) solution. The CF solution, presenting self-quenching concentration of CF, was prepared using 50 mM CF dissolved in 10 mM PBS buffer and 90 mM NaCl, followed by pH adjustment to pH 7.4. CF solution was dispensed on the lipid cake and incubated for 10 min under gentle shaking (50 min-1 on orbital shaker), followed by 1 min vortexing. The liposomes were extruded 21 times with a mini extruder (Avanti Polar Lipids, Inc.), through a 0.1 μm membrane (Nucleapore track-etched hydrophilic membrane). The lipids were purified by gel filtration through a PD MiniTrap G-25 column by elution in PBS buffer (10 mM).

### Carboxyfluorescein release assay

To study peptide efficacy on liposome membrane disruption, the liposome fluorescence was monitored upon peptide addition by microplate reader (Tecan Infinite M1000 Pro, λ_ex_ = 485 nm, λ_em_ = 520 nm). Liposomes were diluted to a concentration of 25 μM with 10 mM PBS buffer and incubated with PLNC8 α, PLNC8 β and PLNC8 αβ at concentration of 10^−5^–10^2^ μM in a 96 well plate at a final volume of 200 μl. Due to the self-quenching concentration of CF inside the liposomes, any fluorescence of the liposome-peptide system could be attributed to the liposome membrane disruption. Fluorescence was monitored prior to peptide addition (F_0_), and continuatively for 30 min. Total CF release (F_T_) was achieved through addition of 1% Triton X-100. The CF release (%) was calculated using the equation:

$$CF_{release}=100\times (F‐F_{0})/(F_{T}‐F_{0})$$

where F is the measured fluorescence intensity at time point t.

### Flavivirus inhibition assay

The antiviral activity of PLNC8 αβ against two flavivirus strains was determined using plaque assay. Crystal violet-based plaque assay was performed to quantify KUNV and immunofocus plaque assay was performed to quantify LGTV. Briefly, a series of virus dilutions in DMEM, untreated or pre-exposed to PLNC8 αβ for 1 h at 37°C, were used to infect semi-confluent Vero E6 cells for 1 h at 37°C, followed by washing and cell-overlaying with DMEM supplemented with 1.2% Avicel (FMC), 2% HI‒FBS (Gibco), 1X non-essential amino acids (Gibco), and 1% PEST (Gibco). After 3 days, the overlays were removed, and cells were fixed by methanol for 20 min before proceeding with the plaque assays. For immunofocus assay, cells were blocked with 2% BSA (Fitzgerald) for 10 min before labeled with mouse anti-E antibody (1:1000, 11H12 anti-TBEV-E, United States Army Medical Research, Institute of Infectious Diseases, Fort Detrick, Frederick, MD, USA), followed by addition of anti-mouse secondary HRP Polymer (1:100) for 1 h at 37°C, and finally KPL TrueBlue Peroxidase Substrate (Seracare) for 15 min at room temperature (RT). For crystal violet-based plaque assay, the fixed cells were stained with 2% crystal violet (Merck, Sigma-Aldrich, Sweden), 20% methanol (Fisher), and 0.1% ammonium oxalate (Sigma) solution. The number of virus plaques was quantified, and representative images of the wells were captured with Olympus SZX9 at 10× magnification.

### SARS-CoV-2 virus inhibition assay

The SARS-CoV-2 virus neutralization assay [24–26] was performed by exposing the viruses (1000 PFU/ml) to PLNC8 αβ in DMEM with 0.1% FCS and PEST for 30 min at 37°C. The peptide-virus mixtures were then transferred to semi-confluent Vero E6 cells and incubated for 1 h. The cells were washed and 2% agarose gel was added together with 200 μl DMEM, 5% FCS. Cell cultures were kept at 37°C for 72–96 h before the assay was terminated. Cells were rinsed with sterile PBS, fixed for 45 min and stained with 2% crystal violet (Merck, Sigma-Aldrich, Sthlm, Sweden) for 45 min. The cells were washed twice with sterile PBS and dried. Plaques were calculated in a plate microscope. All experiment were performed in a biosafety level (BSL)-3 laboratory.

### Influenza A virus inhibition assay

The IAV neutralization assay [27] was performed by diluting PLNC8 αβ in DMEM, supplemented with 0.1% FCS and PEST, followed by incubation with the selected virus dose (1000 PFU/ml) at 37°C for 30 min. The peptide-virus mixtures were then transferred to semi-confluent MDCK cells and incubated for 1 h before washing and addition of 2% agarose gel and 200 μl DMEM-5% FCS. The cells were rinsed with PBS after 72–96 h, fixed for 45 min, and stained with 2% crystal violet for 45 min, followed by washing prior to drying at RT. Plaques were calculated in a plate microscope. The experiment were performed in a BSL-3 facility.

### HIV-1 neutralization assay

The HIV-1 neutralization was performed as previously described [28, 29] using viral isolates derived from HIV-1 subtype B MN strain (http://www.hiv.lanl.gov) in a BSL-3 facility. Antiviral peptides were diluted in RPMI 1640 supplemented with 2% FCS and PEST in 96-well tissue culture plates. Each peptide concentration was mixed with virus at 50 tissue cell-culture infectious dose 50 (50TCID_50_) and incubated at 37°C for 1 h followed by the addition of 10^5^ human PBMCs/0.1 ml and activated by phytohemagglutinin (PHA) and rIL-2 (200–02, PeproTech) or 10^5^ Jurkat T cells/0.1 ml. Cells were incubated for 1.5 h at 37°C and washed twice with RPMI 1640, 5% FCS before receiving new medium. After 3 days, 50% of the medium was exchanged and at 5–7 days of culture, the presence of HIV-1 p24 antigen in the culture medium was measured by capture-ELISA for HIV-1 subtype B. The background of the p24 was determined for each plate and subtracted from all wells. The percentage of neutralization was determined as [1-(mean p24 OD in the presence of test medium/mean p24 OD in the absence of test serum)] × 100.

### Cytotoxicity

Cytotoxicity was determined by measuring the activity of released lactate dehydrogenase (LDH) in the cell culture media upon cell death. Briefly, KUNV suspended in DMEM were either left untreated or exposed to *L*-PLNC8 αβ or *D*-PLNC8 αβ at a final concentration of 10 μM for 1 h. The virus-peptide mixtures were transferred to semi-confluent Vero cells or A549 cells for 1 h, followed by washing and addition of DMEM supplemented with 2.5% FBS. The cells were incubated for 1, 24 and 48 h, and LDH activity was determined using LDH Cytotoxicity Assay Kit (Thermo Scientific™), according to the manufacturers instructions by measuring the absorbance at 490 nm.

### Microscopy for studying membrane disruption

The fluorescent dye Sytox® Green was used to investigate membrane permeabilization caused by PLNC8 αβ. This fluorophore can only cross damaged membranes and fluoresce upon binding to nucleic acids. Briefly, LGTV (10^5^) were suspended in DMEM cell culture medium and were either left untreated or exposed to *L*-PLNC8 αβ, *D*-PLNC8 αβ, or *S*-PLNC8 αβ at a molar ratio of 1:1, or LL-37, for 2 h. The samples were then fixed with 4% PFA followed by addition of Sytox Green for 5 min, and images were captured with Olympus BX41 at 40× magnification. The images were processed and analysed using the software ImageJ [30].

Liposomes containing 5% 2-Oleoyl-1-palmitoyl-sn-glycero-3-phospho-rac-(1-glycerol) (POPG) and with or without cholesterol, were exposed to 1 μM of PLNC8 αβ for 2 min. Cryo-EM specimens were prepared by applying 3 μl liposome mixtures on Quantifoil grids 2/2 coated with a 2 nm carbon layer. Images where collected on a Talos Arctica 200 kV microscope (Thermo Fischer Scientific) using a Falcon3 dorect elelctron detector in linear mode and at a magnification corresponding to a pixel size of 5 Å.

### Statistics

All data were analyzed using GraphPad Prism 9.0 (La Jolla, CA, USA). Data are presented as mean with standard error of the mean (SEM) and analysed using one-way ANOVA with Dunnett´s multiple comparison test (*p<0.05; **p<0.01; ***p<0.001).

## Results

### Susceptibility of flaviviruses and SARS-CoV-2 to PLNC8 αβ

Development of new therapeutics to combat viral infections is considerably valuable to promote human health, and AMPs, including PLNC8 αβ, represent an alternative that needs more consideration. Initially, the antiviral effect of PLNC8 αβ was investigated against LGTV, an attenuated flavivirus that belongs to the tick-borne encephalitis virus (TBEV) complex of the *Flaviviridae* family. Full-length *L*-PLNC8 αβ, but not *S*-PLNC8 αβ or the human cathelicidin-derived peptide LL-37, caused rapid (2 h) and dose-dependent permeabilization of the virus particles shown by Sytox Green staining and fluorescence microscopy (Fig 1A). Quantification of the viral load after exposure of LGTV to the peptides for 1 h showed a dose-dependent decrease by *L*-PLNC8 αβ, resulting in >99% elimination of infective virions, while *S*-PLNC8 αβ and LL-37 did not affect virus infectivity (Fig 1B). Representative images of the cells and virus plaques that are presented above the bars (Fig 1B) show enhanced cell viability due to efficient elimination of virions by *L*-PLNC8 αβ.

**Fig 2:**
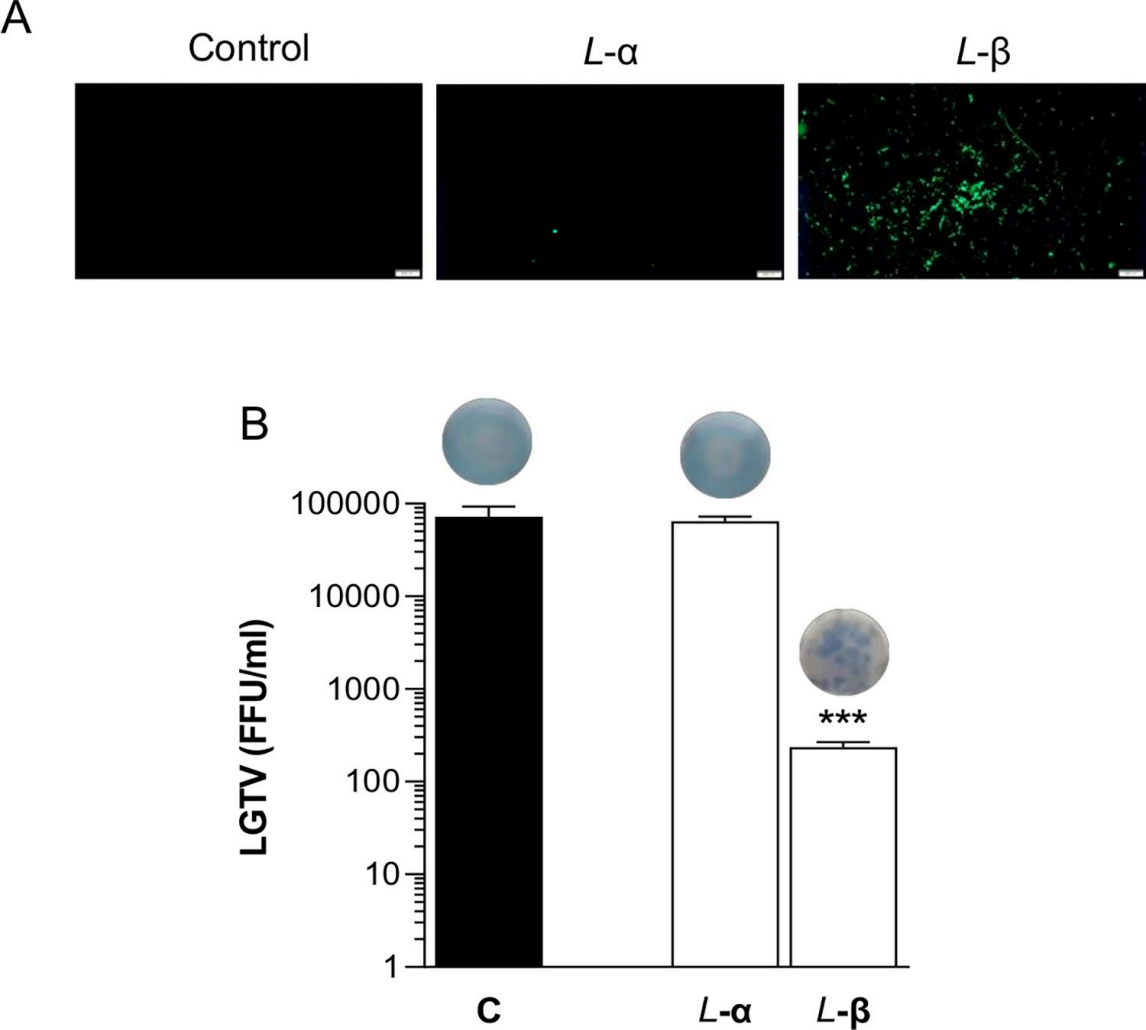
PLNC8 β alone, but not PLNC8 α, shows antiviral properties. LGTV (1×10^5^) were either left untreated or exposed to PLNC8 α or PLNC8 β at a final concentration of 20 μM. **A-** Sytox Green staining, 2 h post-treatment, shows permeabilization of LGTV by PLNC8 β. Scale bar is 200 μm. **B-** LGTV was incubated in cell culture media containing 20 μM of either PLNC8 α or PLNC8 β for 1 h at 37°C, followed by infection of Vero cells for 1 h. Viral load was quantified by performing an immunofocus-based plaque assay. Representative images of the virus plaques are presented above the bars. The virus particles are rapidly permeabilized by PLNC8 β, but not PLNC8 α, and decreased the viral load by >99%. The data (n = 3) are presented as mean with SEM and analysed using one-way ANOVA with Dunnett´s multiple comparison test (***p<0.001).

The results indicate that the antiviral activity of PLNC8 αβ is sequence specific since the effects were completely abolished when scrambling the order of amino acids. Analysis of the contribution of the individual peptides, i.e. PLNC8 α and PLNC8 β, showed that PLNC8 β alone at a final concentration of 20 μM was effective at permeabilizing the virus particles (Fig 2A), and significantly reduced the titer of LGTV (Fig 2B). However, optimal antiviral activity was only achieved when both peptides were present (Fig 1), which significantly lowered the required concentrations to efficiently eliminate infective virions compared to PLNC8 β alone, thus highlighting the importance of PLNC8 α.

**Fig 3:**
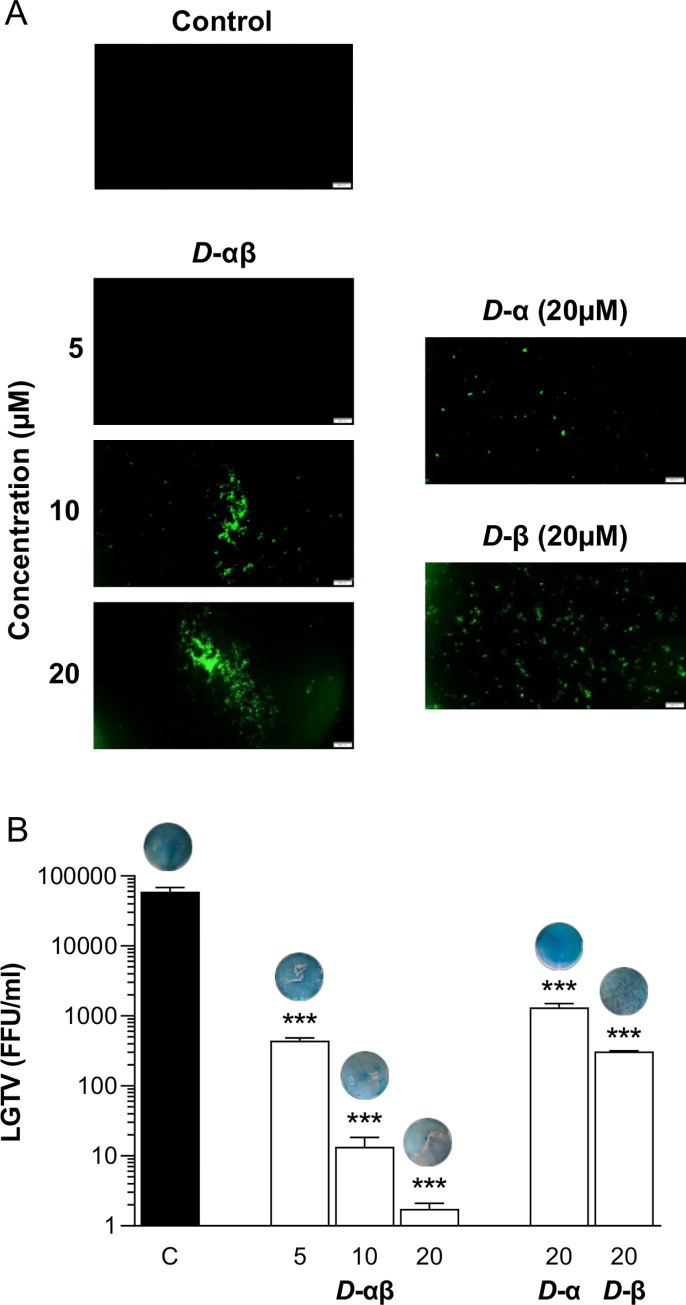
The D-enantiomer of PLNC8 αβ is efficient against the enveloped flavivirus LGTV. Viruses (LGTV, 1×10^5^) were exposed to *D*-PLNC8 α or *D*-PLNC8 β at a final concentration of 20 μM or *D*-PLNC8 αβ (1:1) at a final concentration of 5, 10 and 20 μM. **A**- LGTV were permeabilized by *D*-PLNC8 β and *D*-PLNC8 αβ after 2 h of exposure, while *D*-PLNC8 α was not as efficient, shown by Sytox Green staining. Scale bar is 200 μm. **B**- LGTV was incubated in cell culture media containing the indicated concentrations of *D*-PLNC8 α and *D*-PLNC8 β, either alone or together at a molar ratio of 1:1 for 1 h at 37°C. The suspension was then used to infect Vero cells 1 h followed by quantification of the viral load using immunofocus assay. The indicated concentrations are in μM. Representative images of the virus plaques are presented above the bars. The virus particles are rapidly permeabilized by *D*-PLNC8 αβ, forming large aggregates, and decreased the viral load by >99.9%. *D*-PLNC8 β was more efficient than *D*-PLNC8 α at permeabilizing LGTV. The data (n = 3) are presented as mean with SEM, and analysed using one-way ANOVA with Dunnett´s multiple comparison test (***p<0.001).

These findings encouraged us to determine if the antiviral activity of PLNC8 αβ is mediated through interactions with the lipid membrane or by means of more specific molecular interactions with virion proteins. This was investigated by using the *D*-enantiomers of PLNC8 αβ, which should not be able to bind specifically to the same protein targets as the *L*-enantiomers possibly may bind to. Interestingly, *D*-PLNC8 αβ also showed potent antiviral properties, resulting in rapid permeabilization (Fig 3A) that significantly reduced the titer of infective virions in a dose-dependent manner (Fig 3B). A final PLNC8 αβ concentration of 20 μM resulted in complete elimination of all infective virions after 1 h of exposure. These results indicate that PLNC8 αβ does not bind to a specific protein receptor or structure on LGTV. Furthermore, the contribution of the individual peptides to the obtained antiviral activity of PLNC8 αβ showed that PLNC8 β is more potent than PLNC8 α.

**Fig 4:**
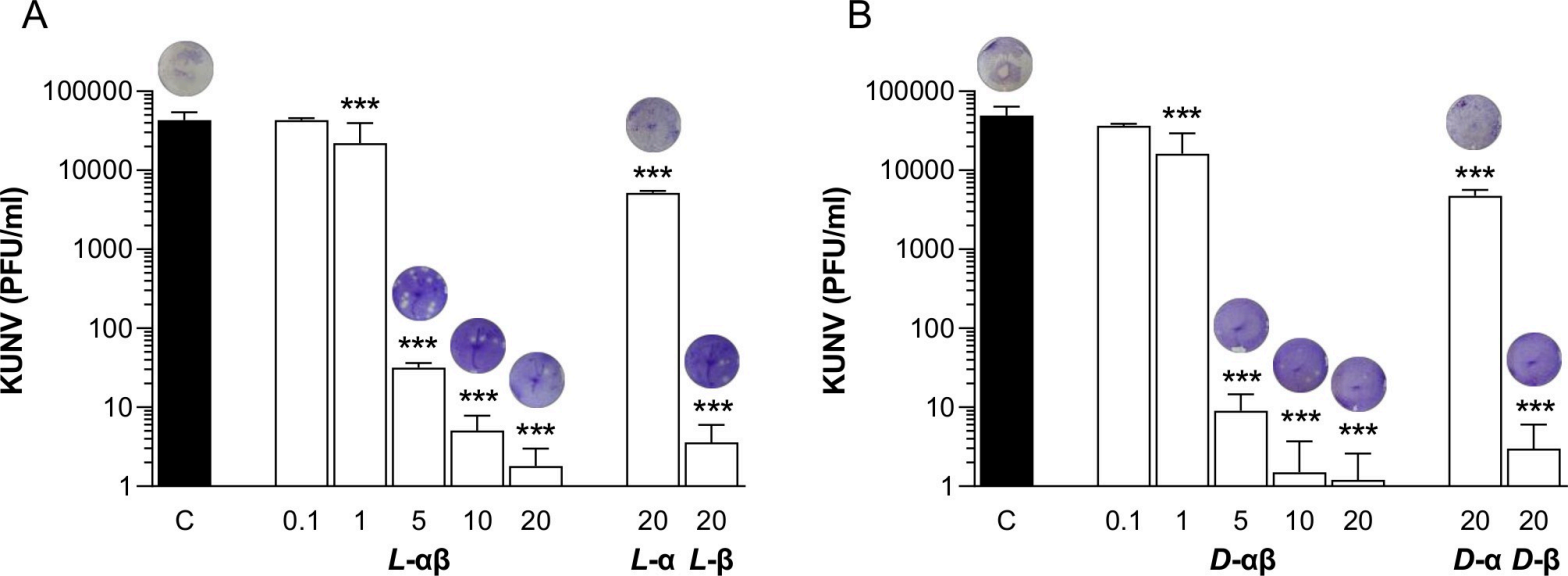
Both L- and D-enantiomers of PLNC8 αβ are effective against WNV_Kunjin_. The flavivirus Kunjin (KUNV, 1×10^5^), suspended in DMEM, was exposed to **A**- L-enantiomer or **B**- D-enantiomer of PLNC8 α or PLNC8 β at a final concentration of 20 μM or PLNC8 αβ (molar ratio 1:1) at a final concentration of 0.1, 1, 5, 10 and 20 μM for 1 h at 37°C. The suspension was then added to Vero cells and incubated for 1 h, followed by quantification of the viral load by performing crystal violet-based plaque assay. The indicated concentrations are in μM. Representative images of the virus plaques are presented above the bars. The virus particles are rapidly permeabilized by both enantiomers of PLNC8 αβ that decreased the viral load by >99.9%. The data (n = 3) are presented as mean with SEM and analysed using one-way ANOVA with Dunnett´s multiple comparison test (***p<0.001).

The antiviral activity of PLNC8 αβ on enveloped viruses was further verified using KUNV, an attenuated variant of West Nile Virus (WNV). Pre-exposure of KUNV to either *L*-PLNC8 αβ (Fig 4A) and *D*-PLNC8 αβ (Fig 4B) for 1 h caused a dose-dependent and significant reduction in the number of infective virions. A final concentration of 5 μM of either *L*-PLNC8 αβ or *D*-PLNC8 αβ caused more than 99.9% reduction in the titer of infective KUNV, concequently promoting cell viability. Similarly, we found that *L*-PLNC8 β and *D-*PLNC8 β alone, respectively, were more efficient than *L*-PLNC8 α and *D*-PLNC8 α at eliminating KUNV (Fig 4A and 4B).

**Fig 5:**
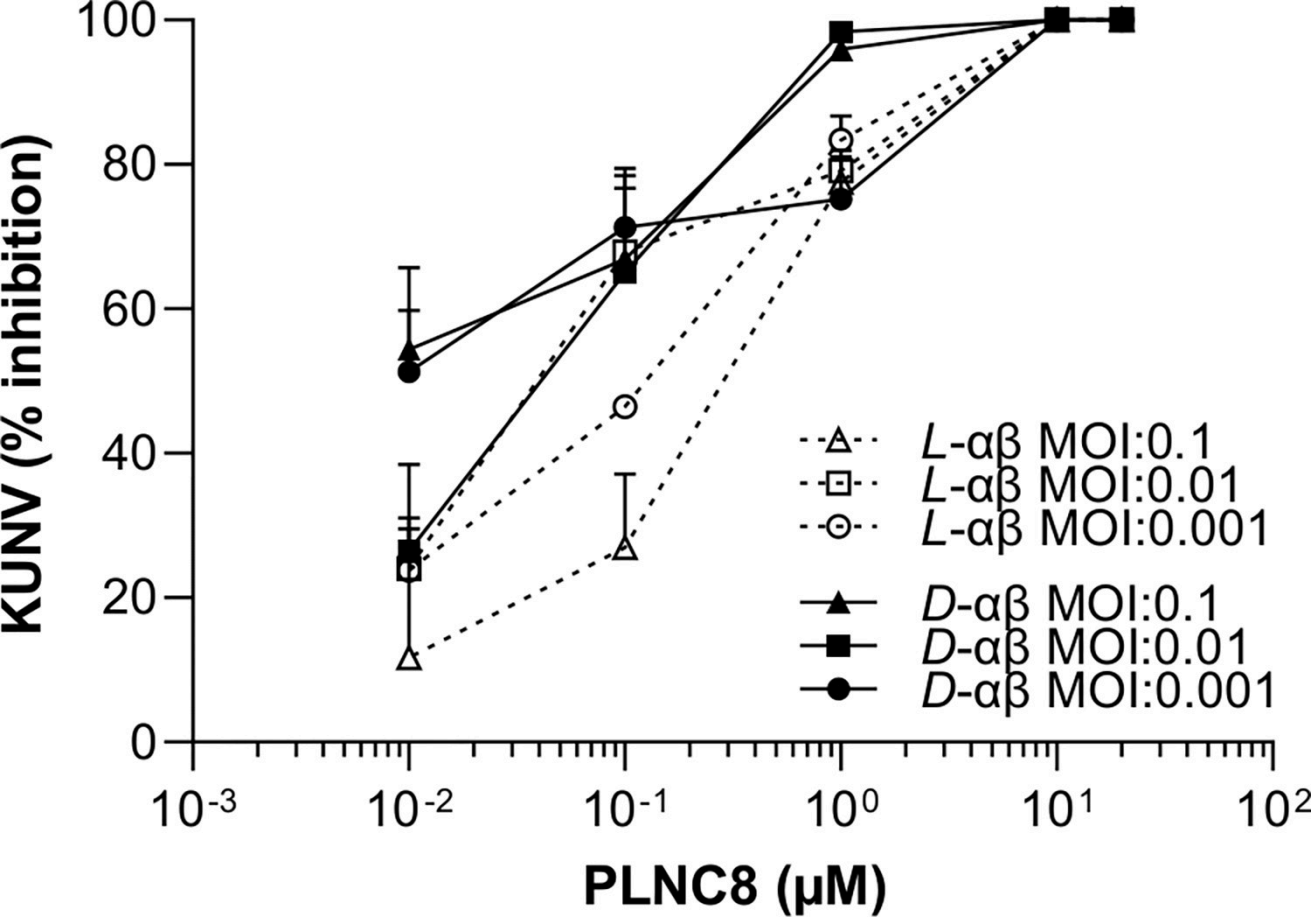
Antiviral activity of L- and D-enantiomers of PLNC8 αβ against different virus titers of WNV_Kunjin_. KUNV, at a multiplicity of infection (MOI) of 0.1 (20000 PFU/ml), 0.01 (2000 PFU/ml), or 0.001 (200 PFU/ml), was exposed to increasing concentrations of either *L*-PLNC8 αβ or *D*-PLNC8 αβ (1:1) for 1 h at 37°C, followed by infection of Vero cells and quantification of the viral load. PLNC8 αβ reduces the viral load even at low concentrations (≤0.1 μM). The data (n = 3) are presented as mean with SEM.

Furthermore, *L*-PLNC8 αβ reduced the viral load of KUNV even at low concentrations (≤ 0.1 μM) (Fig 5). A final peptide concentration of 1 μM reduced the viral load by >50%, while concentrations of ≥10 μM completely eliminated all virions.

**Fig 6:**
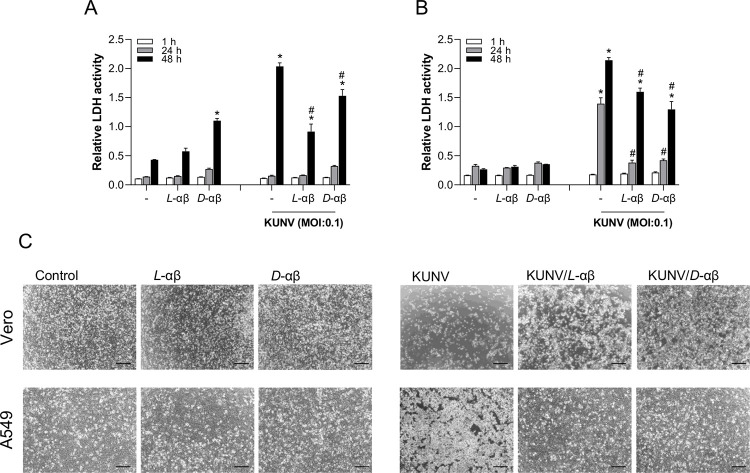
PLNC8 αβ counteracts virus-induced cytotoxicity. KUNV was either left untreated or exposed to 10 μM of *L*-PLNC8 αβ (L-αβ) or *D*-PLNC8 αβ (D-αβ) for 1 h. Relative cytotoxicity of **A**- Vero cells and **B**- human lung epithelial cells (A549) was determined after exposure to PLNC8 αβ, KUNV, or PLNC8 αβ-treated KUNV for 1 h, followed by replacement of the cell culture media to DMEM, supplemented with 2.5% FBS. **C**- representative images of Vero- and A549 cells after 48 h and 24 h of infection/exposure, respectively. Scale bar is 50 μm. The data are presented as mean with SEM, and statistics at one significance level (p<0.05) were analysed using one-way ANOVA with Dunnett´s multiple comparison test (n = 3, significance compared to the unexposed negative control* and KUNV-infected control^#^).

Assessment of cell toxicity in response to PLNC8 αβ and KUNV was determined in Vero cells and the human lung epithelial cell line A549. LDH activity in Vero cells was elevated by *D*-PLNC8 αβ after 48 h (Fig 6A), however the images (Fig 6C) show a confluent layer of cells compared to KUNV that were highly cytotoxic. Both *L*-and *D*-PLNC8 αβ suppressed the cytotoxic effect of KUNV significantly. Human cells were more susceptible to KUNV that caused significant cell death after 24 h, and the peptides were remarkebly efficient in suppressing these effects to basal levels (Fig 6B and 6C). Exposure of human cells to the peptides, upto 48 h, did not cause any cytotoxic effects.

**Fig 7:**
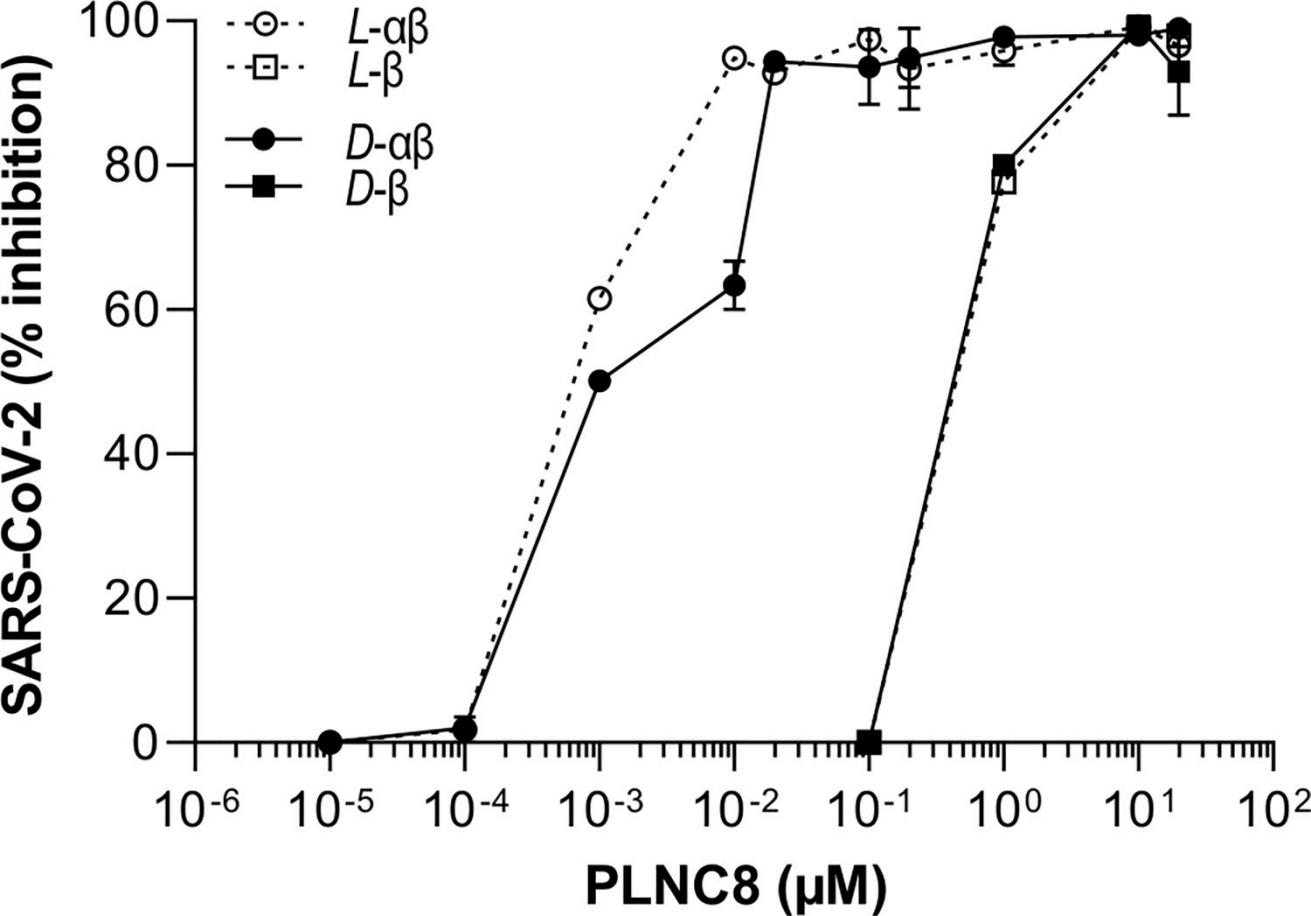
Antiviral activity of PLNC8 αβ against SARS-CoV-2. Viruses (1000 PFU/ml) were exposed to the L-enantiomer or D-enantiomer of PLNC8 β or PLNC8 αβ (1:1) using the indicated concentrations for 1 h at 37°C. The suspension was then used to infect Vero cells and the viral load was quantified by performing plaque assay. Both enantiomers of PLNC8 αβ caused a substantial reduction of the viral load in a dose-dependent manner. The data (n = 4) are presented as mean with SEM.

Our findings of the antiviral properties of PLNC8 αβ prompted us to determine its activity against other enveloped viruses, e.g. SARS-CoV-2. Like flaviviruses, SARS-CoV-2 aquire its envelope from the ER in the infected cell. Both enantiomers of PLNC8 αβ were remarkably efficient against SARS-CoV-2, causing a substantial reduction of the viral load in a dose-dependent manner (Fig 7). A 50% reduction of PFU with the *L*- and *D*-form of PLNC8 αβ was achieved at 0.001 μM. PLNC8 β (*L*- and *D*-form) alone required ~0.5 μM to cause a 50% reduction of the viral load at the same SARS-CoV-2 virus concentration (Fig 7).

### Susceptibility of IAV and HIV-1 to PLNC8 αβ

**Fig 8:**
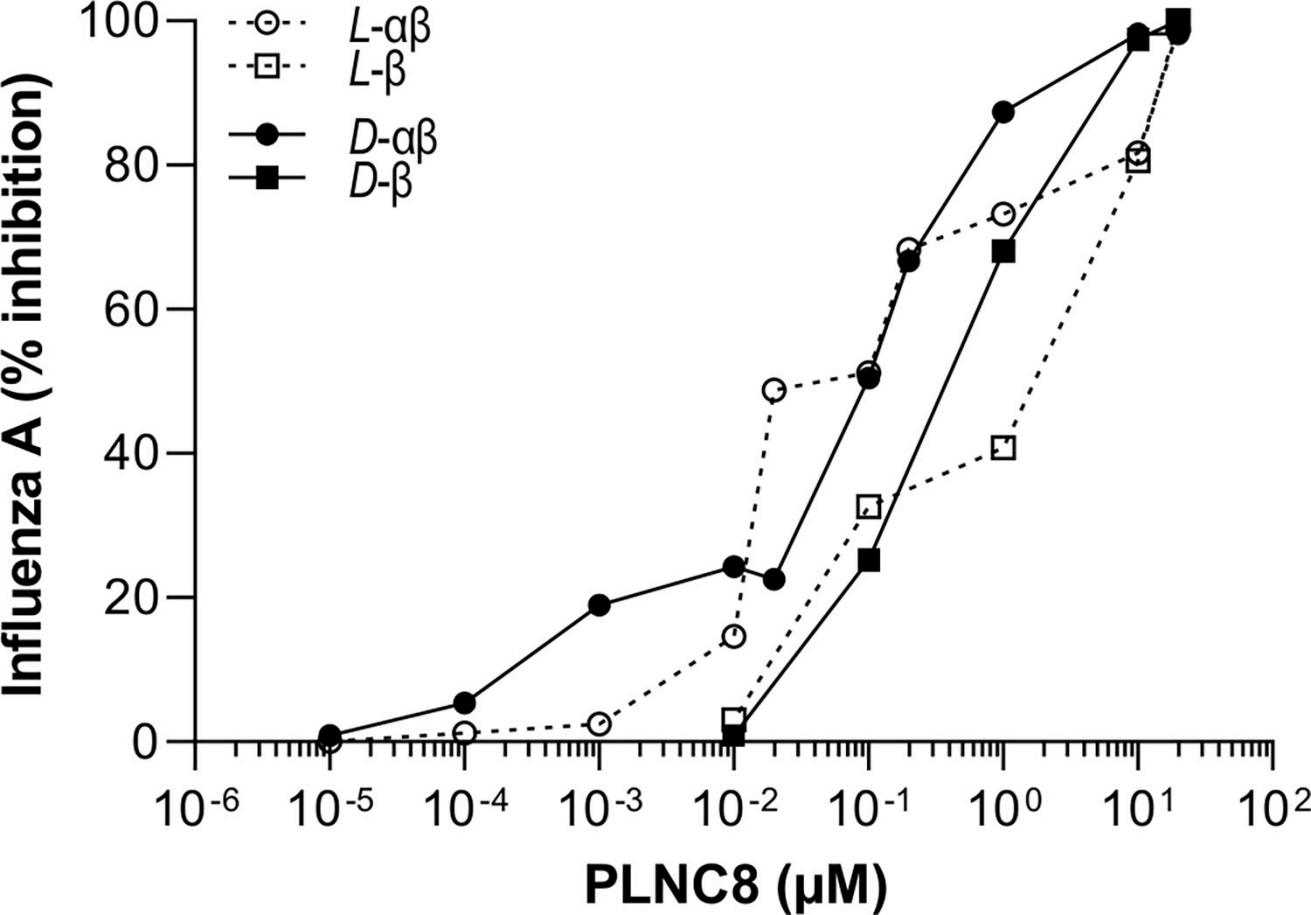
Antiviral activity of PLNC8 αβ against IAV. IAV (1000 PFU/ml) was pre-treated with the L-enantiomer or D-enantiomer of PLNC8 β or PLNC8 αβ (1:1) for 1 h at 37°C. The suspensions were transferred to MDCK cells followed by quantification of the viral load. A final concentration of >0.1 μM of PLNC8 αβ was required to drastically reduce the amount of influenza A. The data (n = 2) are presented as mean.

Since the envelopes of both flaviviruses and coronaviruses are originated from ER/Golgi, we were interested to determine if PLNC8 αβ also was active against viruses that obtain their envelope from the plasma membrane. We tested the antiviral activity of the peptides against IAV and HIV-1. Interestingly, the peptides were less effective against these viruses. A 100-fold higher concentration of *L*-PLNC8 αβ or *D*-PLNC8 αβ was required to reduce IAV by 50% (Fig 8), compared to SARS-CoV-2. A higher concentration of *L*-PLNC8 β alone was required to suppress IAV (1 μM) compared to *L*-PLNC8 αβ. However, *D*-PLNC8 β was more effective than *L*-PLNC8 β and suppressed IAV by 50% at ~0.5 μM.

**Fig 9:**
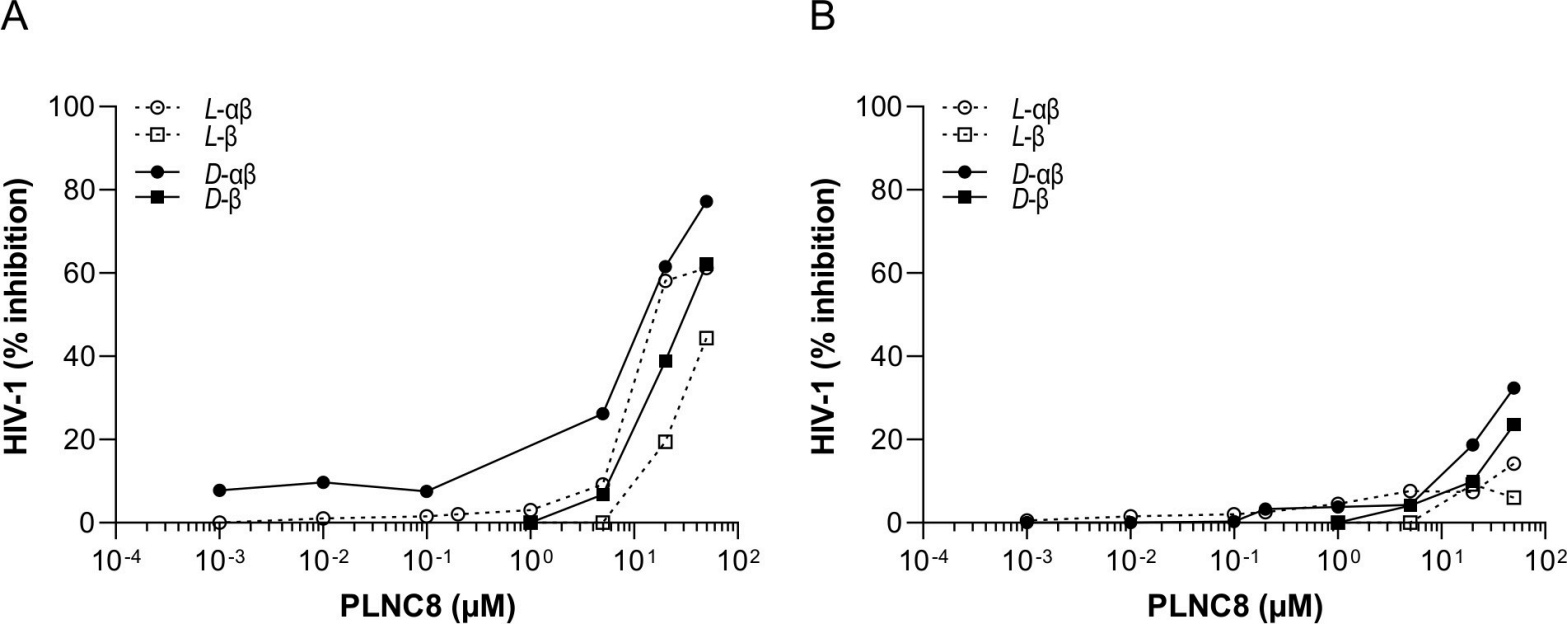
Antiviral activity of PLNC8 αβ against HIV-1. The viruses (50TCID_50_/ml) were treated with the L-enantiomer or D-enantiomer of PLNC8 β or PLNC8 αβ (1:1) for 1 h at 37°C. The suspensions were then transferred to (**A**) Jurkat T-cells or (**B**) isolated primary human PBMC, followed by quantification of the viral load. HIV-1 is more resistant to the membrane perturbing effect of PLNC8 αβ, compared to the other tested viruses. The data (n = 2) are presented as mean.

Moreover, HIV-1 was shown to be more resistant to the membrane perturbing effects of the peptides, compared to the other tested viruses (Fig 9). Interestingly, HIV-1 was more susceptible in Jurkat T-cells, in which the 50% reduction concentration was ~20 μM for both enantiomers of PLNC8 αβ (Fig 9A). In contrast, for human PBMC cells the 50% reduction concentration was not possible to determine at the same HIV-1 virus titer (50TCID50/ml) (Fig 9B) since substantially higher concentrations (>50 μM) are required to eliminate the virions. PLNC8 β showed less active against HIV-1, however, the *D*-enantiomer was more effective than the *L*-enantiomer of PLNC8 β.

### The role of anionic charge in the outer leaflet and cholesterol content of phospholipid membranes in their sensitivity towards PLNC8 αβ

**Fig 10:**
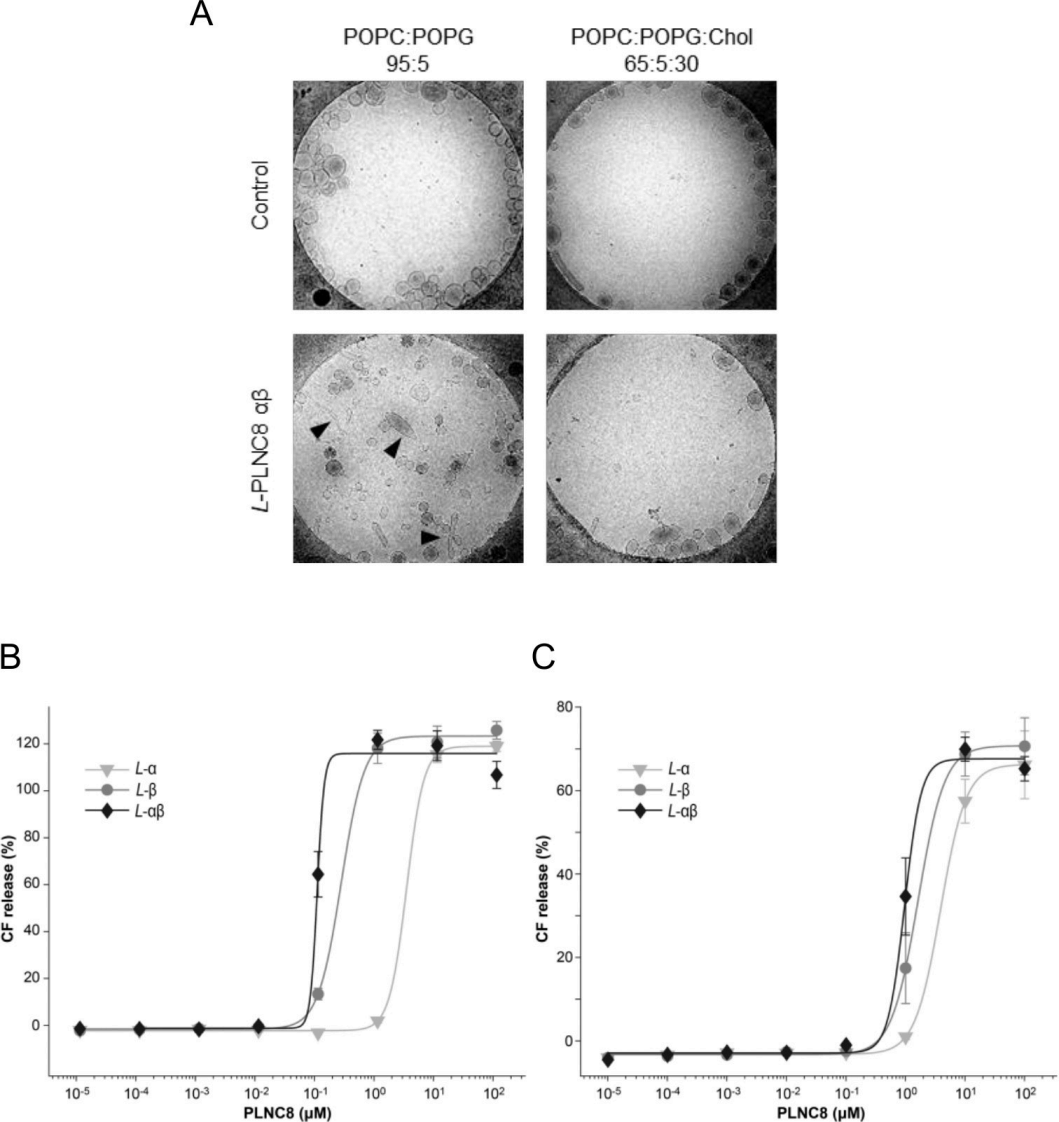
Cholesterol content, phospholipid composition, and total extracellular anionic charge of membranes reflect different viral lipid envelopes and their susceptibility to PLNC8 αβ. **A**- Cryo-EM of liposomes containing 5% phosphatidylglycerol, with or without 30% cholesterol, were either left untreated or exposed to *L*-PLNC8 αβ at a final concentration of 1 μM for 2 min. Liposomes without cholesterol were deformed (black arrowhead) by the peptides and no longer associated with the positively charged carbon on the edges, indicating that the liposomes have lost their negative charge. **B**- Effects of the peptides *L*-PLNC8 α, *L*-PLNC8 β and *L*-PLNC8 αβ on ER-mimetic liposomes containing 81% POPC, 11% POPS and 8% cholesterol, and **C**- plasma membrane mimetic liposomes containing 70% POPC, and 30% cholesterol. The susceptibility of the phospholipid membranes of liposomes to PLNC8 αβ is dependent on the total anionic charge of the outer leaflet and cholesterol content.

The large differences in activity of PLNC8 αβ against the various viruses, depending on whether they acquire their lipid envelopes from the ER or the plasma membrane, encouraged us to investigate these differences using liposomes as a model system. The overall anionic phospholipid charge and cholesterol content of the ER- and plasma membranes, respectively, were mimicked [18, 19]. Interestingly, using cryo-EM, we found that *L*-PLNC8 αβ is able to induce significant deformation of liposomes that did not contain cholesterol, while the cholesterol-containing liposomes were unaffected with a maintained round shape (Fig 10A). The liposome deformation is likely a result of an increased membrane tension upon binding of the peptides to the lipid membrane. The ER lipid membrane model was more susceptible to the membrane perturbing activity of PLNC8 αβ and a final concentration of 0.1 μM was sufficient to completely permeabilize these liposomes (Fig 10B). In contrast, plasma membrane-mimetic liposomes required a 10-fold higher concentration of the peptides to disrupt their integrity (Fig 10C).

## Discussion

Severe virus infections are often life-threatening and emerging epidemic and pandemic virus outbreaks, such as COVID-19, have devastating and global effects on human health. Available antiviral drugs are generally classified into two groups, the first group comprises agents that target viruses at different stages of their life cycle, and the second group comprises agents that target host cell factors [31]. Limitations of most available antiviral agents include emergence of resistance and cytotoxic effects [32, 33]. Development of new broad-spectrum antiviral agents that act on common elements shared by many viruses, independent of their coding genome and thus target viruses irrespectivly of their resistance patterns and mutation rates, would be most advantageous to efficiently combat serious virus infections. AMPs have been studied extensively for their antibacterial activity and the interest to use specific AMPs as antiviral agents has increased significantly [10, 13, 14, 34, 35]. We have previously shown that bacteriocin PLNC8 αβ rapidly and effectively inhibits and kills various bacterial pathogens, e.g., *Staphylococcus* spp., by membrane disruption [16]. The aim of this study was to investigate the membrane perturbing activity of PLNC8 αβ against enveloped viruses.

PLNC8 αβ showed potent antiviral activity against the flaviviruses LGTV and KUNV, and edivence of viral envelope permeabilization was detected microscopically after 2 h. However, this effect probably occurs much earlier, since we have previously shown that the membrane-perturbing effect of PLNC8 αβ is immediate (<2min) [16, 20]. Although PLNC8 β showed antiviral activity by itself, it is obvious that optimal activity is achieved when both peptides are present, thus highlighting the importance of PLNC8 α and the classification of this bacteriocin as class IIb [11, 12, 36]. There are similarities between bacterial membranes and viral envelopes of flaviviruses, including exposed anionic phospholipids that results in a net negative charge on the pathogen surface, which contributes to the initial binding of the cationic PLNC8 αβ peptides to the viral and bacterial surface. The initial viral load (multiplicity of infection, MOI) appears to be an important factor reflecting the activity of the peptides, however not all MOI:s followed this trend. This discrimination may be dependent on incubation time and media composition affecting the availability of the cationic peptides PLNC8 α and β, including formation of micelles/aggregates. A recent report by Strandberg and colleagues [37] showed that phosphate ions cause peptide aggregation that has a detrimental effect on peptide activity. Other factors that can compromize the activity of antimicrobial peptides are cations, including Mg^2+^ and Ca^2+^ [38, 39]. Furthermore, the rapid and efficient elimination of virions by PLNC8 αβ maintains and promotes cell viability, which is important to e.g., establish an adequate inflammatory response towards an infection. However, the use of relevant animal models will be necessary to determine the important pharmacological endpoints adsorption, distribution, metabolism, excretion, and toxicity (ADMET), as well as bioavailability and efficacy of PLNC8 αβ. Peptides are generally reactive and proteolytically unstable compounds, and these characteristics make peptides unsuitable to be administered systemically. Intranasal administration would be a more relevant route to target virus infections affecting the airways.

The exact mechanism of lipid membrane disruption by PLNC8 αβ remains to be determined. However, it has previously been shown that the peptides undergo structural changes in the presence of lipid membranes [40], indicating partitioning-folding coupling. Scrambling the amino acid sequences of PLNC8 α and β completely abolished the antiviral activity. The effects are thus clearly sequence specific, which is commonly seen for other amphipathic membrane active peptides [41, 42]. Morover, we show that PLNC8 αβ does not interact specifically with any protein ligand in flaviviruses since the *D*-enantiomers of PLNC8 α and β induce similar antiviral effects as the *L*-enantiomers. The importance of anionic lipids for efficient membrane disruption by PLNC8 αβ was also shown by using a liposomal model system mimicking the composition of the viral envelope. Binding of PLNC8 αβ is thus likely initiated by electrostatic interactions between these cationic peptides and anionic phospholipids in the viral envelope, followed by peptide folding and lipid membrane insertion.

Since the structure and characteristics of the lipid envelope of flaviviruses are almost the same across all species, the strong antiviral activity of PLNC8 αβ against KUNV and LGTV could probably be extrapolated to other members of the family *Flaviviridae*. These include dengue, zika, and tick-borne encephalitis (TBE) viruses, which are associated with frequent outbreaks in many parts of the world [43]. Similarly, an antiviral activity of PLNC8 αβ would be expected for members of the *Coronaviridae* family. The lipid envelope of flaviviruses is derived from the ER, while coronaviruses exploit the vesicular-tubular trafficking system between the ER and Golgi apparatus, i.e. ER-Golgi intermediate compartments (ERGIC) [44, 45]. The membrane characteristics of the ER and Golgi apparatus are similar, in which both have exposed anionic lipids in their outer leaflets and contain low levels of cholesterol, compared to the much higher content of cholesterol in the plasma membrane [18, 19]. Indeed, we show that both the *L*- and *D*-enantiomers of PLNC8 αβ are remarkably potent against SARS-CoV-2, the virus responsible for the current pandemic. Interestingly, the concentration of PLNC8 αβ required to cause 50% reduction of SARS-CoV-2 in this study is 0.01 μM, which is considerably low compared to other antiviral agents. Li and colleagues showed a virucidal activity of the scorpion venom peptide variant mucroporin-M1 against several RNA viruses, including SARS-CoV with an EC_50_ of 7.12 μM [46], which is more than 1000-fold higher compared to the dose needed for PLNC8 αβ. Based on these results, it is reasonable to suggest that other related *Coronaviridae* viruses, such as Severe Acute Respiratory Syndrome Coronavirus (SARS-CoV) and the Middle East Respiratory Syndrome Coronavirus (MERS-CoV) [47, 48], are susceptible to PLNC8 αβ. This is very interesting from a clinical and public health perspectives, in view of the very limited therapeutic options that are available so far against these viruses.

The difference in antiviral activity of PLNC8 αβ against various enveloped viruses is most likely dependent on the cellular compartment from which the viruses acquire their phospholipid envelope. IAV and HIV-1 gain their lipid envelopes from the plasma membrane and a 100-1000-fold higher peptide concentration is needed to achieve an effect comparable to PLNC8 αβ action on flaviviruses and SARS-CoV-2. IAV was found to be suppressed only after exposure to relatively high levels of PLNC8 αβ (≥1 μM), while HIV-1 was not susceptible even at concentrations up to 50 μM in human PBMC. These results are in line with our previous observations showing that PLNC8 αβ up to 50 μM is not toxic to human cells [16, 20, 49]. This is probably due to that the human plasma membrane has a different composition and orientation of phospholipids, i.e. it is asymmetric and the outer leaflet is composed of zwitterionic phospholipids [18]. Furthermore, the plasma membrane contains a higher proportion of cholesterol compared to the membranes of intracellular organelles, thus providing fluidity and protection [16]. In support, we show in this study that ER-mimetic liposomes are more susceptible to the membrane perturbing effect of PLNC8 αβ compared to the resistant plasma membrane-mimetic liposomes. This is mainly attributed to the beforementioned characteristics, i.e. cholesterol content, phospholipid composition, and anionic charge of the outer leaflet of liposome membranes. However, biological membranes of eukaryotic cells and the structure of mature virions are considerably more complex, and other factors than membrane lipids may contribute to the susceptibility of virions to PLNC8 αβ. This include peptide binding to the spike protein of SARS-CoV-2 and to envelope proteins of flaviviruses, which will be further investigated to unravel the mechanisms of PLNC8 αβ.

This study suggests that the variable susceptibility of different species of enveloped viruses to PLNC8 αβ is primarily dependent on the structure and lipid composition of viral envelopes. The differences between the plasma membrane and the ER membrane are crucial determining factors enabling PLNC8 αβ to efficiently permeabilize and destroy viral envelopes without affecting cell viability. Consequently, these divergences in structure and function between the viral envelope and host cell plasma membrane make viral membranes ideal targets for an antiviral therapy using membrane-active antiviral peptides such as PLNC8 αβ. Indeed, we show that viruses acquiring their lipid envelopes from budding through the ER/Golgi complex pathway, e.g. SARS-CoV-2 and flaviviruses, are considerably more susceptible to PLNC8 αβ, compared to viruses that obtain their lipid envelope from the plasma membrane, such as IAV and HIV-1. This could be explained by the significantly higher cholesterol content in cellular plasma membranes (IAV and HIV-1), and the efficient repair mechanisms in response to damage, which are present in host cells and not the viruses [50].

PLNC8 αβ is a potent antiviral peptide that can potentially be developed as an effective broad-spectrum antiviral drug candidate for prevention and treatment of severe virus infections, primarily for topical applications to target extracellular virions. PLNC8 αβ appears to be less dependent of the virus genetic properties as it mainly targets the lipid compartment of virus envelopes, which is more or less stable since it is derived from membranes of infected host cells. The focus of our future studies will be to investigate the antiviral properties and mechanisms of PLNC8 αβ and optimized derivates on other viruses, including non-enveloped viruses as important controls, with further validation and characterization.
